# Current Hospital Topics

**Published:** 1906-06-30

**Authors:** 


### HOSPITAL ADMINISTRATION.

### CONSTRUCTION AND ECONOMICS.

### CURRENT HOSPITAL TOPICS.

**The Metropolitan Hospital Sunday Fund.**

It has been decided to incorporate this Fund by Royal Charter and the steps taken with this in view have so advanced that there should be no difficulty in securing the charter by the end of the Fund's year—*i.e.* by October 31 next. Much must, of course, depend upon the active co-operation of the Privy Council and its officers, but in all the circumstances we feel sure that this will be cordially extended to the Council with hearty good will.

**Schemes of Amalgamation.**

The King's Fund has done good service by continuously working to secure the amalgamation of certain groups of hospitals. One great difficulty must necessarily be not only to reconcile the conflicting interests of individual institutions but to secure a just basis of amalgamation which shall provide that no wrong is done to the just claims of any officer. Our attention has been recently called to the fact that in regard to one such amalgamation of two hospitals which recently took place, the combined committees dismissed both secretaries without giving to either of them substantial compensation, though both gentlemen had discharged their duties with efficiency and were, as a matter of justice, entitled to the fullest consideration. We mention the matter because it must be apparent to all reasonable people that a mistake of the kind referred to may do more than anything else to render the task of the King's Fund, in bringing about necessary amalgamations, almost, if not quite, impossible for the future, unless care be taken to guard against injustice to individuals. Regulations are needed as a basis of negotiations in all these cases. These should secure that no scheme of amalgamation will be approved in future unless it is so drawn as to prevent its adoption from doing wrong unjustly to any responsible member of the existing staff.

**The Uniform System of Accounts.**

It will be remembered that the King's Fund appointed Mr. J. G. Griffiths, formerly head of the firm of Deloitte, Plender Griffiths and Company, to inquire into and report upon this system with a view to the introduction of certain modifications for the purpose of enabling more accurate comparisons of the expenditure of different hospitals to be made. Mr. Griffiths, to whom the thanks of all the hospitals are due, devoted considerable time and thought to the matter, and his able report is likely to prove most useful in many ways. It is by no means easy, however, to elaborate and perfect a system of accounts for universal adoption by hospitals and great philanthropic institutions which will, in view of continuous developments, meet the views of all concerned. A committee of hospital secretaries has been for some time engaged in perfecting the Index of Classification, which is one vital feature of the uniform system, and we have much pleasure in publishing a letter in another column from Mr. Moore, secretary and superintendent of the Royal Infirmary, Liverpool. It is a great matter, as we recently said, that all the larger hospitals at any rate throughout the country, and indeed throughout the Empire, should keep their books upon the uniform system. We should welcome, therefore, any further correspondence on the subject which is calculated to remove misapprehension and to secure that the revised and perfected system shall, on the whole, fulfil the purposes for which it has been elaborated.

**Hospital Management in Victoria.**

The colony of Victoria and the whole hospital world has experienced a great loss by the death of Mr. F. W. Haddon, formerly editor of the *Melbourne Argus*. He was the active spirit with Mr. A. Cornish in preparing a very valuable report on the Melbourne Hospital some years ago, and the *Melbourne Argus* raised a fund for that institution of £20,000. One of the last acts of his life was to write a further report in which he endeavours to show why the Melbourne Hospital should be considered to hold a position different from all others in Victoria. Mr. Haddon commenced by stating that the cost per bed at the Melbourne Hospital (1903-4) on the daily average of beds occupied was £73 2s. 6d., while at the Alfred Hospital, the next largest and most important general hospital in Victoria, the cost per bed was only £55 12s. 9d. Mr. Haddon maintained that the Melbourne Hospital holds a quite exceptional position, because it is the great medical and surgical institution not only for Melbourne, but for the whole Colony. He next proceeded to analyse the classes from which the patients are drawn, followed them to their homes, classified their diseases, and concluded that the cases treated are not only difficult and expensive, medically and surgically, but that the vitality of the patients is often so low that they have to be fed up at considerable cost to the hospital. So great is the pressure of the patients seeking admission that the ordeal of selection for a bed is in practice unusually severe. Mr. Haddon maintains that there are absolutely no light and cheap cases to be found at the Melbourne Hospital; all are severe and more or less expensive. The Melbourne Hospital "is the only hospital properly equipped for the treatment of septic cases, and the consequence is that the large proportion of such cases are sent there." Mr. Haddon then proceeds to draw a comparison beween the Melbourne Hospital and the Alfred Hospital. He shows that, whereas the Melbourne Hospital has 64 and the Alfred Hospital 36 per cent. of the total hospital beds available, the Melbourne Hospital treats 68 per cent. and the Alfred Hospital only 32 per cent. of the in-patients relieved. At the former hospital each bed was occupied by fifteen patients during the year, while at the latter the number of occupants was only 12½. At the former hospital the average residence of each patient was 24.5 days as compared with 29.5 days at the latter. Mr. Haddon then proceeded to attempt a comparison between the number of cases of a more serious kind treated at the two hospitals and to make comments on the figures and facts he produces. It is difficult to follow Mr. Haddon here, as a great deal more might be said, and further and fuller facts are needed before an outsider can pass judgment upon them. Mr. Haddon's report has not unnaturally aroused a good deal of discussion in the Colony, and at the instance of the managers of the Alfred Hospital Mr. E. C. Norman, its secretary and superintendent, has presented a report in reply to certain statements recently made by the Premier of Victoria. We have not space to quote this report which is able and convincing as far as it goes. We withhold comment upon it because we must first obtain the Premier's speech and his reply to the report in question. There can be no doubt that British hospitals would be rendered a great service if someone would take up the rôle adopted by Mr. F. W. Haddon, in Victoria, and follow it out with persistence and zeal in this country at the present time.

